# Depatuxizumab Mafodotin (Depatux-M) Plus Temozolomide in Recurrent Glioblastoma Patients: Real-World Experience from a Multicenter Study of Italian Association of Neuro-Oncology (AINO)

**DOI:** 10.3390/cancers13112773

**Published:** 2021-06-03

**Authors:** Marta Padovan, Marica Eoli, Alessia Pellerino, Simona Rizzato, Claudia Caserta, Matteo Simonelli, Maria Michiara, Mario Caccese, Elena Anghileri, Giulia Cerretti, Roberta Rudà, Vittorina Zagonel, Giuseppe Lombardi

**Affiliations:** 1 Department of Oncology, Oncology 1, Veneto Institute of Oncology IOV-IRCCS, 35128 Padua, Italy; marta.padovan@iov.veneto.it (M.P.); mario.caccese@iov.veneto.it (M.C.); giulia.cerretti@iov.veneto.it (G.C.); vittorina.zagonel@iov.veneto.it (V.Z.); 2 Unit of Molecular Neuro-Oncology, Besta-IRCCS, 20133 Milan, Italy; marica.eoli@istituto-besta.it (M.E.); elena.anghileri@istituto-besta.it (E.A.); 3 Department of Neuro-Oncology, University and City of Health and Science Hospital, 10126 Turin, Italy; alessia.pellerino@unito.it (A.P.); roberta.ruda@aulss2.veneto.it (R.R.); 4 Department of Oncology, Azienda Sanitaria Universitaria Friuli Centrale, 33100 Udine, Italy; simona.rizzato@asufc.sanita.fvg.it; 5 Medical Oncology Unit, Azienda Ospedaliera S. Maria, 05100 Terni, Italy; caserta_claudia@libero.it; 6 Department of Biomedical Sciences, Humanitas University, 20090 Pieve Emanuele, Italy; matteo.simonelli@hunimed.eu; 7 IRCCS Humanitas Research Hospital, 20089 Rozzano, Italy; 8 Medical Oncology Unit, University Hospital of Parma, 43126 Parma, Italy; Michiara@ao.pr.it; 9 Department of Neurology, San Giacomo Hospital, 31033 Castelfranco Veneto, Italy

**Keywords:** glioblastoma, EGFR, Depatux-M, antibody drug conjugate, targeted therapy

## Abstract

**Simple Summary:**

Depatux-M is an antibody-drug conjugate against activated EGFR. The efficacy and tolerability of the Depatux-M and temozolomide combination in recurrent glioblastoma patients were recently analyzed in the INTELLANCE-2/EORTC 1410 phase 2 trial. Despite the trial was negative, it showed interesting results for patients received this combination therapy versus standard treatment. For the first time worldwide, we investigated this treatment in a real-life population. Interestingly, we reported encouraging clinical benefits close to that reported in the previous randomized INTELLANCE 2 trial. Ocular toxicity was manageable. Likely, a subgroup of patients could benefit of this treatment and so, significant molecular predictors of treatment efficacy such as EGFR SNVs should be better investigated in a larger prospective study.

**Abstract:**

Background: Depatuxizumab Mafodotin (Depatux-M; ABT-414) is an antibody-drug conjugate consisting of a specific antibody against activated EGFR and a cytotoxic agent with antimicrotubule activity. The INTELLANCE 2/EORTC 1410 phase 2 trial produced interesting results for the combination regimen of Depatux-M and temozolomide in EGFR-amplified glioblastoma patients at first recurrence. For the first time worldwide, our work investigated the clinical outcome and safety of this combination in a real-life population. Materials and Methods: Patients were enrolled from seven AINO (Italian Association of Neuro-Oncology) Institutions. The major inclusion criteria were: histologically confirmed diagnosis of glioblastoma, EGFR-amplified, one or more prior systemic therapies and ECOG PS ≤ 2. According to the original schedule, patients received Depatux-M 1.25 mg/kg every 2 weeks combined with temozolomide. The primary endpoints of the study were overall survival and safety. Results: A total of 36 patients were enrolled. The median age was 57 years, ECOG PS was 0–1 in 28 patients (88%), MGMT methylated status was found in 22 (64%), 15 patients (42%) received the combined treatment as second-line therapy. The median OS was 8.04 months (95% CI, 5.3–10.7), the 12 month-OS was 37%. On univariate and multivariate analyses, the MGMT methylation status was the only factor resulting significantly associated with survival. Grade 3 ocular toxicity occurred in 11% of patients; no grade 4 ocular toxicity was reported. No death was considered to be drug-related. Conclusions: The study reported the first “real world” experience of Depatux-M plus temozolomide in recurrent glioblastoma patients. Encouraging clinical benefits were demonstrated, even though most patients were treated beyond second-line therapy. Overall, the results are close to those reported in the previous phase 2 trial. Toxicity was moderate and manageable.

## 1. Introduction

Glioblastoma (GBM) is the most common and aggressive primary brain tumor. While chemoradiotherapy with temozolomide (TMZ) represents standard first-line therapy after surgery [1], the standard of care for second-line treatment has not been defined. Treatment options include re-surgery, re-irradiation and systemic pharmacotherapy—mostly nitrosoureas [2,3]. Bevacizumab demonstrated that it prolongs progression-free survival (PFS) without improving overall survival (OS) [4]; yet, immunotherapy with checkpoint inhibitors showed no efficacy in glioma patients [5,6].

Some recent studies have improved knowledge on molecular pathways and specific gene mutations driving the origin and progression of GBM; we are therefore seeing some promising results due to specific targeted therapies (i.e., BRAF inhibitors, TRK inhibitors and regorafenib) [2,7,8], even though the benefit of these new treatments needs further evaluation.

EGFR (epidermal growth factor receptor) is a transmembrane tyrosine kinase involved in many intracellular pathways regulating DNA synthesis and cell proliferation. Its signaling abnormalities have a prominent role in the pathogenesis of GBM; approximately 50% of GBM patients have tumors harboring EGFR amplification on chromosome 7 and half of these cases have the exon 2–7 deletion, known as EGFR variant III (EGFRvIII) [9]. However, many EGFR tyrosine kinase inhibitors (TKIs) such as gefitinib [10], erlotinib [11] and afatinib [12] failed to demonstrate efficacy in both newly-diagnosed and recurrent GBM.

Depatuxizumab Mafodotin (Depatux-M; ABT-414) is an antibody-drug conjugate composed of the anti-EGFR monoclonal antibody conjugated to monomethyl auristatin F (mafodotin), an antimicrotubule agent [13]. After interaction with the tumor-specific binding site (exposed and available in the case of EGFR amplification), Depatux-M is internalized in the cell and mafodotin is released, thus killing the cell [14]. Depatux-M was initially evaluated in the phase 1 M12-356 (NCT01800695) trial [15]; this study analyzed the safety, preliminary efficacy and pharmacokinetics of Depatux-M alone or in combination with TMZ in patients with newly-diagnosed or recurrent GBM. The combination arm showed interesting results in the recurrent GBM population, with an objective response rate of 14.3% and a 6-month OS of 69.1%. A subsequent phase 2 study was planned on the basis of this promising data: INTELLANCE 2/EORTC1410 (NCT02343406) was a multicenter, randomized open label study comparing Depatux-M plus TMZ (arm 1) versus Depatux-M alone (arm 2) versus standard treatment of TMZ/lomustine (arm 3/control arm) in EGFR-amplified GBM at first recurrence/progression after standard chemoradiotherapy [16]. Although the study did not reach the primary endpoint of OS, at the time of long-term follow-up the combination arm demonstrated a statistically longer OS compared to the other arms. Worth noting among patients treated with the combination regimen is that the benefit in OS was more consistent for those who relapsed/progressed more than 16 weeks after the start of the last cycle of TMZ. The Depatux-M toxicity profile was similar to the phase 1 study’s observed toxicities.

We performed this multicenter study in order to better evaluate the safety and efficacy of this combination regimen in the real-life population of recurrent GBM patients.

## 2. Results

A total of 36 patients were enrolled from seven Institutions of the Italian Association of Neuro-Oncology (AINO) from October 2018 to June 2019. Baseline patient and tumor characteristics are shown in Table 1: the median age was 57 years, the ECOG PS was 0–1 in 88% of patients, 42% received the treatment as second-line therapy and 27% underwent further chemotherapy at subsequent progression. Most patients presented IDH 1–2 wild type status (94%) and methylated MGMT promoter (64%). The median number of Depatux-M infusions was 4 (range 1–24). Most patients (81%) started Depatux-M plus TMZ more than 16 weeks since the last TMZ cycle. No patient received surgery after the experimental treatment.

**Figure 1:**
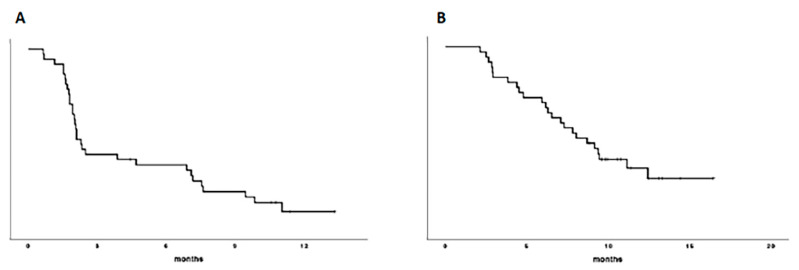
Median PFS (**A**) was 2.1 months (95% CI, 1.7–2.4) and the 6 month-PFS was 38%; median OS (**B**) was 8.04 months (95% CI, 5.3–10.7) and the 12 month-OS was 37%.

At the time of analysis, 24 patients (67%) had died and 31 patients (86%) had progressed. Median PFS was 2.1 months (95% CI, 1.7–2.4) and the 6 month-PFS was 38% (Figure 1A); median OS was 8.04 months (95% CI, 5.3–10.7) and the 12 month-OS was 37% (Figure 1B); Figure 1. PFS (A) and OS (B) curves.

On univariate and multivariate analysis, the MGMT methylation status was an independent prognosticator for longer OS (see Table 2 and Table 3); ECOG PS, number of prior lines of therapy and the time starting Depatux-M since TMZ were not significantly correlated to OS.

None of the factor studied were significantly correlated to PFS on multivariate analysis.

One (3%) out of 36 patients and 4 (11%) out of 36 patients had a complete response and a partial response, respectively, with an objective response rate of 14% (see Table 4). Additionally, 13 patients (36%) had stable disease, resulting in a disease control rate (DCR) of 50%. No clinical or molecular factors were associated with DCR (see Table 5).

The most frequent Depatux-M related adverse events (AEs) were ocular and were observed in 81% of patients (any grade); in most cases the ocular events occurred after the second administration of Depatux-M. Ocular AEs were attributed to microcystic keratopathy and included keratitis (67%), photophobia (8%), eye pain (3%) and conjunctivitis (3%). Grade 3 keratitis was reported in four cases (11%) and no grade 4 adverse events were recorded (see Table 6). Hematological toxicities (mainly thrombocytopenia) were reported in 17 patients (47%) and in six cases (17%) were classified as grade 3–4. Grade 3 hypertransaminasemia was recorded in one patient (3%), two cases (6%) documented as grade 3 venous thrombosis and grade 1–2 hypertension was reported in only one case (3%).

Ocular toxicity led to Depatux-M dose delay and dose reductions in 28% and 17% of patients, respectively, while its early permanent discontinuation occurred in two patients (5% of cases). No drug-related death was reported.

## 3. Discussion

To our knowledge, this is the first study to assess the role of Depatux-M plus TMZ in recurrent EGFR-amplified GBM patients in a real-life world. Clinical benefits were very close to those reported in the combination therapy arm in the previous phase 2 trial. The INTELLANCE 2 protocol was a randomized multicentric study which analyzed the efficacy of Depatux-M alone versus Depatux-M plus TMZ versus the standard treatment of lomustine or TMZ in EGFR-amplified GBM patients relapsing after the Stupp protocol [16]. The primary analysis of the INTELLANCE 2 study with a median follow-up of 14.4 months (199 subjects had died) showed a trend of longer survival for the combination regimen versus the standard treatment: the 12m-OS rate was 39.7% versus 28.2%, respectively, with a *p* value of 0.06 (HR = 0.71, 95% CI, 0.50–1.02); in the subsequent long-term analysis with a median follow-up of 28.7 months (237 patients had died), the OS difference between the two arms became statistically significant: the 2-year survival in the combination arm was 19.8% versus 5.2% in the control arm (*p* = 0.017; HR = 0.66, 95% CI, 0.47–0.93). Interestingly, in INTELLANCE 2, the MGMT methylation status was not statistically associated with a longer OS while the clinical benefit of the Depatux-M plus TMZ combination was more consistent in patients relapsing more than 16 weeks after the end of TMZ treatment. Conversely, we reported a statistically longer OS in patients with MGMT promoter methylated tumors and no survival advantage for patients starting the treatment after 16 weeks since TMZ; this could be due to the smaller population analyzed in this real-world study, although the presence of methylated MGMT can represent a significant predictor of TMZ efficacy [17]. Moreover, more patients with methylated MGMT were enrolled in our study compared to the INTELLANCE 2 trial: 61% versus 48.9%, respectively. Yet, the high rate of MGMT methylated patients, as well as the high rate of patients who started treatment more than 16 weeks after the end of TMZ, may have contributed to the clinical benefit observed in the present study.

Even though most of the patients in our study were treated beyond second-line therapy (58%), we reported a similar OS to the prior randomized INTELLANCE 2 study; indeed, 58% of patients received the therapy as a third or subsequent line of treatment. Yet, our results were similar or even superior to those reported in some larger phase 2 or 3 studies analyzing the recurrent GBM population. In the EORTC 26101 trial [4], 437 patients were treated with lomustine plus bevacizumab or lomustine alone, reporting a 12m-OS rate of 31.5% and 34.1%, respectively. In the TAMIGA trial [18], 123 patients with recurrent GBM after receiving radiation therapy, plus bevacizumab and TMZ as a first-line treatment, were randomized to receive bevacizumab plus lomustine or lomustine alone: the 12m-OS rate was 11.7% and 16.5%, respectively. Yet, the randomized phase 2 REGOMA trial [7] showed a statistically longer OS in recurrent GBM patients when treated with regorafenib compared to standard lomustine: the 12m-OS rate was 38.9% versus 15.0%, respectively. Due to this important clinical benefit, regorafenib was included in the NCCN 2020 guidelines and was approved by the Italian Medicines Agency (AIFA) as the preferred treatment for the recurrent GBM population [2].

Worth noting in this real-word study analyzing the efficacy and safety of Depatux-M plus TMZ outside the context of randomized controlled trials is that we demonstrated the benefit of this combination regimen, also in terms of disease control rate. Indeed, we reported a DCR of 50% (one patient with complete response, four with partial response and 13 with stable disease), which is again very close to that observed in INTELLANCE 2 (48% of evaluable cases). Hovewer, an important limit of our study is the relatively small number of included cases and this could impact the results of our “real-life” analysis both in terms of tolerability and efficacy.

As regards safety, similar to INTELLANCE 2, the most common adverse events were ocular and reported in 81% of patients compared to the 87% observed in the phase 2 study. However, we observed grade 3 ocular toxicity (all cases of keratitis) in only 11% of patients (no grade 4 ocular toxicity was recorded) versus 40% observed in the prior phase 1 study [15] (mostly keratitis in 13% of cases) and 32.9% shown in INTELLANCE 2 trial. Different factors may have contributed to the lower incidence of severe ocular toxicity observed in our study. An increased expertise regarding the management of Depatux-M has been gained by neuro-oncologists which have previously participated in the INTELLANCE 1 and 2 trials; another key factor contributing the management of ocular toxicity is that all patients enrolled in our study were followed by a dedicated ophthalmologist. Indeed, it was already demonstrated that the ocular side effects caused by Depatux-M can be reversible and moderate if carefully managed [19,20]. In our study, Depatux-M dose reduction occurred in 17% of patients versus 12% reported in the previous phase 1 study, while dose delays occurred in only 28% versus the 58% seen in the prior study [15]. The lower rate of patients having dose delays may also have a positive impact on survival.

The addition of Depatux-M to standard radiation and TMZ in newly-diagnosed EGFR-amplified GBM patients was investigated in the randomized phase 3 INTELLANCE 1 trial. This trial was recently stopped after an interim analysis due to a lack of survival benefit for patients receiving Depatux-M compared with the placebo when added to the standard therapy. Despite this recent failure, based on our data and on the INTELLANCE 2 long-term analysis, the combination regimen may be effective in a more favorable subgroup of recurrent EGFR-amplified GBM patients. About that, recent post-hoc detailed molecular analyses on patients treated within the INTELLANCE 2/EORTC 1410 phase 2 clinical trial demonstrated that tumors harboring EGFR single-nucleotide variants (SNVs) have improved survival in the Depatux-M plus TMZ combination arm [21]; EGFR mutations result in a receptor that is hypersensitive to ligand and increase transformation towards the active conformation of the protein. Unfortunately, we did not perform these molecular analyses to confirm the predictor role of EGFR SNVs on treatment efficacy.

## 4. Materials and Methods

### 4.1. Study Design and Patients

This was a single arm, multicenter observational study undertaken in 7 AINO (Italian Association of Neuro-Oncology) clinical centers that were involved in the Abbvie pre-approval access (PAA) program to Depatuxizumab Mafodotin for patients with recurrent GBM. Patients eligible for inclusion were those with histologically-confirmed GBM, with unequivocal first or subsequent disease progression after receiving standard radiochemotherapy. Other major inclusion criteria were: EGFR amplification locally assessed by fluorescence in situ hybridization (FISH) or by real-time quantitative PCR on tumoral tissue at first surgery; an age of at least 18 years; an Eastern Cooperative Oncology Group (ECOG) performance status (PS) score of 2 or lower (or Karnofsky performance score ≥ 70); evidence of disease progression on brain MRI as defined by Response Assessment in Neuro-Oncology (RANO) criteria [22]; prior systemic therapies (≥1) were allowed with the exclusion of EGFR targeting agents. Surgery at the time of recurrence was acceptable. Eligibility criteria also included an adequate hematological, renal and hepatic function. The clinical data of patients receiving Depatux-M plus TMZ were entered prospectively into medical records.

### 4.2. Study Procedures

Patients received Depatux-M 1.25 mg/kg by IV infusion over 40 min on day 1 and day 15 and TMZ 150–200 mg/m^2^ on days 1–5 of a 28-day cycle. Prophylactic steroid eye drops were administered for 7 days, from 2 days before until 4 days after Depatux-M. Dose adjustments of TMZ and Depatux-M were allowed according to the INTELLANCE 2/EORTC1410 protocol [16]; in particular, Depatux-M must be reduced in case of first grade 3 toxicity (after recovery to grade 1) and could continue at 0.75 mg/kg or 0.5 mg/kg in case of repeated grade 3 toxicity. To monitor ocular toxicity, ophthalmology examinations were performed at baseline and after 2 weeks from the start of treatment. Subsequent ophthalmological evaluation, steroid eye drops and other supportive care measures (lubricating eye drops, local antibiotic, therapeutic bandage, etc.) were routinely used to control ocular symptoms when clinically indicated. Toxicities were graded using the Common Terminology Criteria for Adverse Events, Version 5.0.

Clinical evaluation, serum chemistry and hematological parameters were assessed at baseline and every two weeks during treatment. Radiological assessment was carried out at baseline by gadolinium brain MRI and then every eight to twelve weeks until disease progression or when clinically indicated. Neuroradiological response was evaluated by the local Investigator in accordance with RANO criteria.

The EGFR amplification status was locally assessed in each Institution by means of fluorescence in situ hybridization (FISH) or real-time quantitative PCR on tumoral tissue [14] at first surgery. EGFR was considered amplified in FISH when there is focal EGFR gene amplification defined as an EGFR/CEP 7 (chromosome enumeration probe 7) ratio greater than or equal to 2 in ≥15% of cells [14]. The evaluation of MGMT methylation was performed by pyrosequencing or methylation-specific PCR and isocitrate dehydrogenase (IDH) 1/2 mutational status by immunohistochemistry or by Sanger sequencing.

### 4.3. Outcomes and Statistical Analysis

The primary endpoints were OS and safety. OS was defined as the time from the start of treatment until death from any cause. Secondary endpoints were PFS, defined as the time from the start of treatment to progression of tumor or death), the rate of patients achieving disease control (DCR) as best response according to RANO criteria during the treatment (disease control = patient reporting stable disease or partial response or complete response), and objective response rate (ORR; objective response = patient reporting partial response or complete response). PFS and OS were evaluated using the Kaplan–Meier survival curves and the log-rank test was used for group comparisons. Patients without PFS or OS were censored at last assessment.

A multivariate Cox proportional hazards regression model was used for multivariate analysis to test the effect of prognostic factors in terms of OS and PFS. A univariate inclusion criterion of *p* ≤ 0.2 was used for the multivariate model. Categorical variables were compared using Fisher’s exact test. *p* values were based on 2-side testing and differences with a *p* ≤ 0.05 were considered significant. Adverse events and laboratory abnormalities were reported by the worst grade experienced by the patient.

All statistical analyses were performed using SPSS Version 26 statistical software (SPSS Inc., Chicago, IL, USA).

## 5. Conclusions

In conclusion, to our best knowledge this is the first study that have investigated the combination treatment of Depatux-M plus TMZ in a real-life population of recurrent EGFR-amplified GBM. Although, most of our patients received the treatment beyond the second-line, we observed a substantial clinical benefit close to that reported in the previous randomized INTELLANCE 2 trial. Ocular toxicity was frequent but manageable. Lastly, significant molecular predictors of treatment efficacy such as EGFR SNVs should be better investigated in a larger prospective study.

## Figures and Tables

**Table 1:** Baseline patients and tumor characteristics.

Variables	Number (%)
**Median age** (range)	57 (38–73)
**Gender**	
Male	25 (69%)
Female	11 (31%)
**ECOG PS**	
0–1	28 (88%)
2	8 (12%)
**MGMT status**	
Methylated	23 (64%)
Unmethylated	13 (36%)
**IDH 1–2 status**	
Mutated	1 (3%)
Wild Type	34 (94%)
Not available	1 (3%)
**Line of treatment**	
2nd line	15 (42%)
>2nd line	21 (58%)
**Start of Depatux-M since TMZ**	
>16 weeks	29 (81%)
≤16 weeks	7 (19%)

ECOG PS: Eastern Cooperative Oncology Group Performance Status; MGMT: O-6-methylguanine-DNA methyltransferase; IDH: Isocitrate dehydrogenase; TMZ: temozolomide.

**Table 2:** Univariate analysis for PFS and OS.

Variables	Subgroups	PFS		OS	
		Median (ms)	*p*	Median (ms)	*p*
MGMT status			**0.05**		**0.02**
	Met	2.3		11.7	
	Unmet	2.004		6.5	
ECOG PS	0–1	2.1	0.5	9.3	0.1
	2	2.1		7.09	
Line of Depatux-M treatment			0.14		0.3
	2nd line	2.3		9.4	
	>2nd line	2.04		7.1	
Start of Depatux-M since TMZ			0.14		0.45
	≤16 wks	4.6		9.1	
	>16 wks	2.1		8.04	

ECOG PS: Eastern Cooperative Oncology Group Performance Status; MGMT: O-6-methylguanine-DNA methyltransferase; TMZ: temozolomide; PFS: progression-free survival; OS: overall survival; the statistically significant variables are in bold.

**Table 3:** Multivariate analysis for PFS and OS.

Variables	PFS		OS	
	HR (95% CI)	*p*	HR (95% CI)	*p*
MGMT status	0.6 (0.2–1.2)	0.1	0.3 (0.1–0.9)	**0.03**
ECOG PS	-	-	1.7 (0.6–4.8)	0.3
Line of treatment	0.9 (0.3–2.5)	0.9	-	-
Start of Depatux-M since TMZ	2.2 (0.3–2.5)	0.14	-	-

ECOG PS: Eastern Cooperative Oncology Group performance status; MGMT: O-6-methylguanine-DNA methyltransferase; TMZ: temozolomide; PFS: progression-free survival; OS: overall survival; the statistically significant variables are in bold.

**Table 4:** Response evaluation (RANO criteria).

Type of Response	N (%)
Complete response	1 (3%)
Partial response	4 (11%)
*Objective response*	5 (14%)
Stable disease	13 (36%)
*Disease control*	18 (50%)
Progressive disease	18 (50%)

**Table 5:** Factors correlating with disease control.

Variable	χ^2^	*p* Value
MGMT	1.4	0.2
ECOG PS	0.04	0.8
Line of treatment	0.3	0.53
Start of Depatux-M since TMZ	0.34	0.2

ECOG PS: Eastern Cooperative Oncology Group performance status; MGMT: O-6-methylguanine-DNA methyltransferase; TMZ: temozolomide; χ^2^: chi-squared test.

**Table 6:** Drug-related treatment-emergent adverse events.

Adverse Event	Grade			
	1	2	3	4
Eye disorders	5 (14%)	20 (56%)	4 (11%)	0
*Keratitis*	5 (14%)	15 (42%)	4 (11%)	0
*Photophobia*	0	3 (8%)	0	0
*Eye Pain*	0	1 (3%)	0	0
*Conjunctivitis*	0	1 (3%)	0	0
Hematology	7 (19%)	4 (11%)	4 (11%)	2 (6%)
Hypertransaminasemia	1 (3%)	1 (3%)	2 (6%)	0
Hypertension	1 (3%)	0	0	0
Venous Thrombosis	0	0	2 (6%)	0

## Data Availability

The data were not publicly archived.
